# Glypican Gene GPC5 Participates in the Behavioral Response to Ethanol: Evidence from Humans, Mice, and Fruit Flies

**DOI:** 10.1534/g3.111.000976

**Published:** 2011-12-01

**Authors:** Geoff Joslyn, Fred W. Wolf, Gerry Brush, Lianqun Wu, Marc Schuckit, Raymond L. White

**Affiliations:** * Ernest Gallo Clinic and Research Center, Emeryville, California 94608; † Department of Psychiatry, University of California, San Diego, San Diego, California 92161; ‡ Department of Neurology, University of California, San Francisco, San Francisco, California 94143

**Keywords:** alcoholism, response to alcohol, glypican, behavior

## Abstract

Alcohol use disorders are influenced by many interacting genetic and environmental factors. Highlighting this complexity is the observation that large genome-wide association experiments have implicated many genes with weak statistical support. Experimental model systems, cell culture and animal, have identified many genes and pathways involved in ethanol response, but their applicability to the development of alcohol use disorders in humans is undetermined. To overcome the limitations of any single experimental system, the analytical strategy used here was to identify genes that exert common phenotypic effects across multiple experimental systems. Specifically, we (1) performed a mouse linkage analysis to identify quantitative trait loci that influence ethanol-induced ataxia; (2) performed a human genetic association analysis of the mouse-identified loci against ethanol-induced body sway, a phenotype that is not only comparable to the mouse ethanol-ataxia phenotype but is also a genetically influenced endophenotype of alcohol use disorders; (3) performed behavioral genetic experiments in *Drosophila* showing that fly homologs of GPC5, the member of the glypican gene family implicated by both the human and mouse genetic analyses, influence the fly’s response to ethanol; and (4) discovered data from the literature demonstrating that the genetically implicated gene’s expression is not only temporally and spatially consistent with involvement in ethanol-induced behaviors but is also modulated by ethanol. The convergence of these data provides strong support to the hypothesis that GPC5 is involved in cellular and organismal ethanol response and the etiology of alcohol use disorders in humans.

The etiology of alcohol use disorders (AUD) involves the interaction of many genetic, environmental and behavioral factors (Bierut 2011; Goldman *et al.* 2005; Kimura and Higuchi 2011). Two approaches have been taken to reduce this complexity and make the genetics of AUDs more experimentally tractable. Animal and cell models provide the simplest, most flexible experimental systems, but their relevance to the human disorder is a concern. In humans, efforts have been made to develop endophenotypes; simpler, intermediate phenotypes predictive of AUD development but more directly influenced by genetic variation (Gottesman and Gould 2003). One prominent AUD endophenotype is a low level of response (LR) to ethanol. Low LR is genetically influenced (Heath and Martin 1992; Madden *et al.* 1995; Martin *et al.* 1981) and predicative of later AUD development (Quinn and Fromme 2011; Schuckit and Smith 2000), and independent studies have demonstrated genetic association of alcohol dependence and ethanol LR to several polymorphisms, including the same nicotinic receptor locus (Joslyn *et al.* 2008; Wang *et al.* 2008).

Recent genome-wide association (GWA) studies have highlighted the genetic complexity of AUDs. Large surveys involving hundreds to thousands of subjects identified a single intergenic locus marked by rs7590720 that barely reached statistical significance after correcting for multiple tests (Bierut *et al.* 2010; Edenberg *et al.* 2010; Joslyn *et al.* 2010; Treutlein *et al.* 2009). A recent meta-analysis of over 45,000 individuals uncovered a different marker, rs6943555 in the AUTS2 gene, which just reaches statistical significance (Schumann *et al.* 2011). Such weak association results that explain a very small proportion of the heritability have been observed in other genetically influenced behavioral disorders. This has been interpreted to mean that the population genetic susceptibility is either expressed through a large number of alleles common in the population that individually confer a very small risk and/or alleles that confer a larger individual risk but are rare in the population and are thus not captured by association with the common SNPs used in GWA studies (Manolio *et al.* 2009; Yang *et al.* 2010). Theoretically, discovering such susceptibility alleles will require very large (hundreds of thousands) subject samples, combined with whole genome sequencing, to enable the genotyping of rare alleles. In practice, such surveys are years in the future: the samples must be collected; sequencing technology needs to further mature; and analysis methods need to be further developed.

Cell and animal models used to evaluate the genetic contributions to AUD risk also face interpretational challenges. Cell culture models have demonstrated that gene expression and cellular signaling are altered by exposure to alcohol [reviewed by Moonat *et al.* (2010)]. While such *in vitro* studies enable experimental dissection of cellular ethanol response, it is not clear that the cellular stress response to a sublethal dose of ethanol shares mechanisms with human AUDs. Rodent models have been widely used because these animals can be taught complex alcohol-related behaviors that can then be manipulated and evaluated by pharmacological, genetic, and electrophysiological techniques [reviewed by Lovinger and Crabbe (2005) and Stuber *et al.* (2010)]. However, the applicability of the rodent behaviors to the human condition is difficult to establish. Invertebrate models of ethanol response, specifically *Drosophila melanogaster* and *Caenorhabditis elegans*, afford greater genetic manipulability than rodents (Wolf and Heberlein 2003), enabling detailed genetic pathways influencing the behavior to be constructed. However, even more so than rodent experiments, relating invertebrate behaviors to human ethanol response is challenging.

In this article, we use a cross-species experimental analysis approach, reasoning that if multiple experimental models suggest the same mechanism, we have increased our chances of discovering an etiologically significant component of AUDs. To this end, we first analyzed mouse and human genetic data identifying a candidate gene putatively involved in ethanol response in both species. We then gained further experimental support for the candidate gene by determining whether functionally disruptive alleles of the *Drosophila* homologs alter the fly’s response to ethanol. Through this approach, we identified GPC5, a member of the glypican gene family. Glypicans are well characterized as modulators of developmental signaling pathways, such as Wnts, Hedgehogs, and Bone Morphogenic Proteins [reviewed by Selleck (2000)]. Intriguingly, evidence is now accumulating that these same pathways participate not only in neural development but also in synaptic maintenance and plasticity, leading to the hypothesis that they are involved in the expression of behavioral phenotypes (Budnik and Salinas 2011; Farías *et al.* 2010; Ille and Sommer 2005; Marqués 2005; Okerlund and Cheyette 2011; Salinas 2005).

## Materials and Methods

### Phenotype selection

Finding a phenotype that is related to alcohol dependence (AD) and comparable across humans, mice, and flies was central to this analysis. While AD cannot be accurately modeled in mice and flies, acute response to ethanol ingestion is similar between these organisms, and in humans, alcohol response is predictive of later AUD development (Erblich and Earleywine 1999; Pollock 1992; Quinn and Fromme 2011; Schuckit and Smith 1996; Schuckit and Smith 2000; Schuckit *et al*. 2000; Schuckit *et al*. 1996). We compared locomotor responses to ethanol: body sway in humans, ataxia in mice, and locomotor activity in flies.

### BXD ethanol-induced ataxia QTL interval mapping

BXD recombinant inbred mouse data were retrieved and analyses were performed using the WebQTL (www.genenetwork.org) data and analysis suite (Wang *et al.* 2003). GeneNetwork is an online data repository with integrated computational tools designed to allow users to explore complex genetic networks by integrating data from different sources. The BXD resource (a small subset of the collected data) contains over 2800 measured phenotypes, which were contributed by hundreds of scientists, all linked to genotypes, providing an easily accessible tool to genetically map traits of interest. Phenotypes were selected by searching the BXD phenotype database using the search strings “ethanol AND ataxia” as well as “alcohol AND ataxia,” From the returned list of phenotypes, those that described ataxic response to the administration of ethanol were selected for interval mapping.

Ethanol-induced ataxia QTL were mapped using the Interval Mapping module. A likelihood ratio statistic (LRS) was calculated for each marker using 1000 permutations. LRS values were converted to the log of the odds (LOD) ratio to be consistent with human genetic reporting norms: LOD = LRS/4.61.

### Mouse human synteny and marker selection

Mouse chromosomal intervals yielding suggestive linkage for an ethanol-induced ataxia phenotype had their human syntenic counterpart identified using data collected and presented by the comparative genomics functions of Ensembl (www.ensembl.org). The proximal- and distal-most markers exhibiting suggestive linkage defined the limits of mouse ethanol ataxia QTL. Using Ensembl’s mouse genome reference NCBIM37, all genes, either wholly or partially within the defined QTL, were identified. Using the Ensembl comparative genomics module, the syntenic human genes (genome reference GRCh37) were identified. Human SNPs were selected for analysis if located within a syntenic gene as defined by its transcript plus 100 kb of flanking sequence. All analyzed genotypes were derived from the Illumina HumanCNV370-Duo DNA Analysis BeadChip.

### Subjects

The human subjects genotyped and phenotyped in this study are part of the San Diego Sibling Pair investigation. Subjects were collected under a protocol approved by the Human Subjects Protection Committee of the University of California, San Diego (UCSD) and is described in greater detail elsewhere (Schuckit *et al.* 2005; Wilhelmsen *et al.* 2003). Participants, ages 18–25, were selected from UCSD students who responded to a randomly mailed questionnaire and who met the following criteria: (1) had a minimum family size of two siblings, male or female, 18–25 years old; (2) had consumed alcohol but had NEVER BEEN alcohol dependent; (3) had at least one parent who met the criteria for alcohol dependence using the *Diagnostic and Statistical Manual of Mental Disorders* (DSM-IV-TR) (American Psychiatric Association 2000); (4) had never met criteria for antisocial personality disorder or any DSM-IV Axis I psychiatric condition. In addition to collecting further questionnaire data, selected subjects were given an alcohol challenge in a laboratory setting to measure their responses to an approximately 0.75 ml/kg of ethanol consumed within 8–10 min (dose was weight- and sex-adjusted to produce similar blood alcohol levels). Body sway was measured using a harness attached to the chest at the level of the axilla from which two perpendicular ropes extended forward and to the left side, passing over pulleys that measured the number of centimeters of movement per minute as gathered through three 1 min evaluations at each time point (Schuckit and Gold 1988). Anterior-posterior body sway (BSA) at the time of peak alcohol effect (60 min) was tested for genetic marker association in our analyses. To reduce ethnic heterogeneity, only Caucasian subjects were analyzed. In total, 367 subjects, 134 males and 233 females, were selected from 186 independent families: 38 singleton families; 121 two-sibling families; 23 three-sibling families; 3 four-sibling families; and a single six-sibling family. The actual number of subjects per marker-phenotype analysis varied slightly because of missing genotype and phenotype data.

Subjects were not consented for release to public databases. Data may be available for sharing under a confidential agreement between a requestor and the authors.

### DNA preparation and genotyping

DNA was extracted from blood specimens within 5 days of the draw using Gentra Puregene reagents and protocols (http://www1.qiagen.com). Extracted DNA was quantified using the Pico Green method (Molecular Probes/Invitrogen), and all DNA solutions were normalized to a common concentration for genotyping assays. Genotyping was carried out using the Illumina HumanCNV370-Duo DNA Analysis BeadChip. Genotype generation and quality control were performed by deCODE Genotyping Service (http://www.decode.com/).

### Human association analysis

Because BSA is not normally distributed (skewness and kurtosis: 1.29 and 2.28), it was corrected for non-normality using the Box-Cox transformation (Box and Cox 1964; Venables and Ripley 2002). The scores were then scaled to mean = 0 and SD = 1 to make them comparable essentially as Z-scores.

Association tests were performed in R (R Development Core Team 2011) with the lmekin function of the kinship package (Atkinson and Therneau 2008). This function provides a linear mixed-effects model whereby the genetic relatedness among individuals (based on the kinship coefficient) is incorporated into the covariance structure of the random effects. This adjusts the model fit and compensates for the fact that the siblings are related and, therefore, so are their genotypes and phenotypes, which would otherwise violate the assumption of independent observations in a linear regression model.

The fixed-effects portion of the model was used for testing the association between a single SNP and BSA. The SNP was treated in R as a factor with three levels (categories), which is similar to coding the major homozygotes as 1/1, the heterozygotes as 0/1, and the minor homozygotes as 0/0. For each SNP, two tests were performed to determine if a given genotype class differed in its average phenotype from the other genotypes. The test compared (a) the major homozygotes (1/1) and the heterozygotes (0/1), and (b) the major homozygotes (1/1) and the minor homozygotes (0/0). Tests (a) and (b) were performed by testing the significance of the regression coefficients from zero, of the heterozygote term and the minor homozygote term, while holding the major homozygote coefficient constant at zero. For rare SNPs, minor homozygote individuals are often not present in the sample, in which case, only test (a) was available for the SNP. In all cases, the Wald test was used to examine the significance of the regression coefficients.

The large number of tests performed in the analysis required that the nominal *P*-values be adjusted for multiple testing. For this, false discovery rate (FDR) q-values were calculated using the method described by Storey and Tibshirani (2003).

### *Drosophila* behavioral experiments

*Drosophila* strains were maintained on standard cornmeal/molasses/yeast media at 25°C and 70% humidity with an approximately 16-hr/8-hr light/dark schedule. All strains were outcrossed for five generations to the Berlin genetic background strain carrying the *w^1118^* eye-color marker. Strain sources were *dally^MB950^* (*MB00950*: Bloomington Stock Center), *dally^80^* (Xinhua Lin), *dlp^f03537^* (Exelixis/Harvard), and *dlp^1^* (Scott Selleck).

Ethanol-stimulated locomotor activity and sedation kinetics were determined as previously described (Kong *et al.* 2010; Rothenfluh *et al.* 2006; Wolf *et al.* 2002). Ethanol vapor was mixed with a humidified air stream at set ratios to achieve a final flow rate of 5.5 L/min and was delivered to individual exposure chambers, each containing a group of 20 genetically identical flies. For locomotor activity, flies were filmed just prior to and during a 25 min exposure to 47% ethanol vapor. For sedation kinetics, flies were exposed to 67% ethanol vapor, and the number of flies that lost the ability to right themselves was counted at 3 min intervals. All behavioral experiments were repeated on separate days with flies derived from separate crosses.

Quantitative PCR was carried out on reverse-transcribed RNA from fly heads according to the manufacturer’s instructions (Applied Biosystems) on an ABI PRISM 7900 Sequence Detection System, using expression levels of RpL32 as a standard to normalize sample concentrations. TaqMan probesets (Applied Biosystems) used in this study were *dally*: Dm01822385_g1, *dlp*: Dm01798599_g1, and *RpL32*: Dm02151827_g1.

Ethanol absorption was measured by exposing groups of 25 flies to either ethanol vapor (47%) or humidified air for 30 min. Flies were immediately frozen on dry ice, and the ethanol concentration in whole-fly homogenates was measured with an alcohol dehydrogenase–based spectrophotometric assay (Diagnostic Chemicals, Ltd.).

## Results

**Figure 1:**
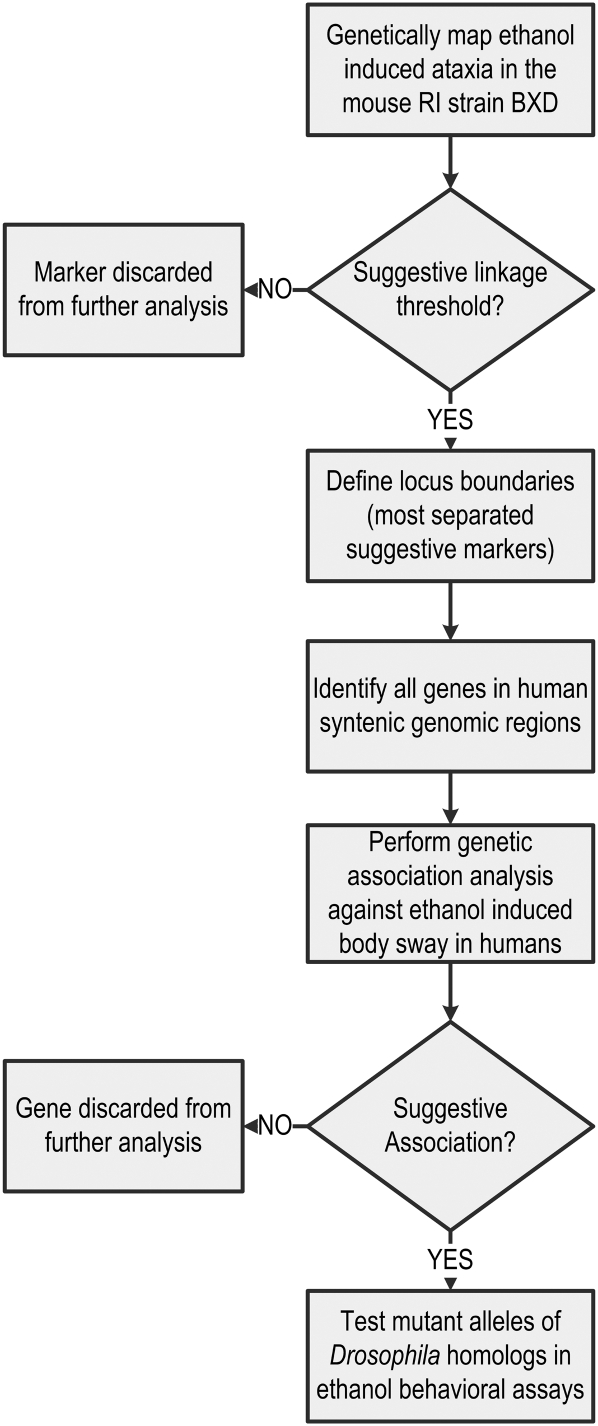
Flowchart of the experimental strategy leading to the identification of GPC5 as a participant in ethanol-induced behaviors.

Data analysis followed the logic presented in Figure 1. First, mouse ethanol response QTL were mapped in the recombinant inbred strain BXD, a strain constructed from the parental strains C57/B6 and DBA, which has been extensively phenotyped for alcohol-related behaviors. We limited our analysis to ethanol-induced ataxia as our ultimate goal was to compare the BXD result with human data. Ethanol-induced ataxia is a phenotypic measure held in common between mouse and human. All BXD data and QTL interval mapping were performed using the tools available on the genenetwork.org website as described in *Materials and Methods*. Twelve phenotypes comparable to the human body sway phenotype were present in the GeneNetwork database and were analyzed (Table 1).

**Table 1:** BXD phenotypes analyzed for genetic linkage

Article PMID	GeneNetwork ID	Description
10656187	10042	Ethanol response (2.5 g/kg ip), ataxia, screen test sensitivity measured as the latency to fall, saline response minus ethanol response [seconds] by K. E. Browman and colleagues
6683363	10078	Ethanol response (dose, ip), ataxia on grid test, 2 to 10 min after injection [errors/run] by J. C. Crabbe and colleagues
8627537	10144	Ethanol sensitivity, initial ethanol-induced ataxia, onset threshold [mg/kg] by E. J. Gallaher and colleagues
8627537	10145	Ethanol response (dose route), maximal threshold to ethanol-induced ataxia [mg/ml] by E. J. Gallaher and colleagues
12183685	10347	Ethanol response (1.75 g/kg ip), initial sensitivity measured by blood ethanol concentration (BEC, retrobulbar bleed) at loss of balance using a dowel test (BEC time 0) [mg % ethanol] by S. L. Kirstein and colleagues
12183685	10349	Ethanol response (1.75 mg/kg ip), time to ataxia measured as loss of balance using a dowel test (Loss corresponds to BEC time 0) [min] by S. L. Kirstein and colleagues
12183685	10350	Ethanol response (1.75 mg/kg ip), duration of ataxia following the first ethanol injection using a dowel test (Regain Test 1 corresponds to BEC1) [min] by S. L. Kirstein and colleagues
8627538	10497	Ethanol response (2 g/kg ip), acute ataxia, difference between day 3 (first ethanol treatment) and day 2 (saline baseline) in the chronic drug group [n grid test errors/10 min test] by T. J. Phillips and colleagues
8627538	10498	Ethanol response (2 g/kg ip), acute ataxia measured using grid test (Accuscan activity monitor), difference between day 11 (first and only ethanol treatment) and day 2 (saline baseline) in the chronic saline group [n errors/activity counts/10 min test] by T. J. Phillips and colleagues
8627538	10500	Ethanol response (2 g/kg ip), difference in ataxia using grid test (Accuscan activity monitor) between acute ethanol on day 3 (first ethanol treatment) and day 2 (saline baseline) in the chronic ethanol group [n errors/activity counts/10 min test] by T. J. Phillips and colleagues
8627538	10501	Ethanol response (2 g/kg ip), difference in ataxia using grid test (Accuscan activity monitor) between injection on day 11 (first and only ethanol treatment) and day 2 (saline baseline) in the chronic saline group [errors/activity counts/10 min test] by T. J. Phillips and colleagues
18830130	11006	Ethanol response (1.75 g/kg ip), ataxia measured by rotarod performance, difference from saline baseline (supplementary data to Brigman *et al.*) by J. L. Brigman and colleagues

Data can be retrieved at genenetwork.org using the GeneNetwork ID. The PMID links to the primary publication describing the experimental protocol (www.ncbi.nlm.nih.gov/pubmed/).

Ethanol-induced ataxia QTL were mapped using the Interval Mapping module of GeneNetwork. LRS/LOD scores were calculated using 1000 permutations. The algorithm defines two statistical thresholds, *suggestive* and *significant*, as defined by Lander and Kruglyak (1995). While no *significant* linkages were discovered, 10 of the 12 phenotypes defined 13 *suggestive* ethanol-induced ataxia QTL (Table 2).

**Table 2:** Summary of BXD linkage and human association of the syntenic locus

Mouse Chromosome	Phenotype	Max LOD	Human Markers Tested	Maximum Associated Marker	Association q-value	Human Genes
14	11006	3.88	476	rs1330469	0.0820	GPC5
1a	10144	2.72	1343	rs790022	0.1360	EFHD1
						ALPI
	10147	2.48				CHRND
						CHRNG
2	10144	2.43	635	rs6077309	0.1510	ANGPT4
						RSPO4
						FAM110A
3	10498	2.92	335	rs16828127	0.2240	NLGN1
11c	10347	2.41	333	rs9892427	0.2695	ELAC2
18b	10500	2.29	639	rs11875845	0.3970	DCC
1b	10146	2.53	509	rs10515988	0.4040	ZCCHC2
11a	10078	3.36	361	rs9885172	0.6060	SLIT3
6	10042	2.91	244	rs2375016	0.6230	SVOP
10	10500	2.21	163	rs7397470	0.6630	TBK1
						XPOT
18a	10147	2.09	704	RS2684847	0.7040	DTNA
11b	10042	3.89	319	RS9892427	0.9800	GRB7
						ERBB2
						NEUROD2
						TCAP
						STARD3
						PNMT
						PPP1R1B
13	10498	2.21	154	rs7716600	0.9950	MRPS30
	10500	1.96				
	10501	1.96				

All loci with “suggestive” linkage to at least one BXD ethanol-induced ataxia phenotype are listed. The first three columns describe the BXD linkage (see Table 1 to decipher phenotype code). If a mouse chromosome has more than one linkage signal, the loci are differentiated with a letter after the chromosome number. The next four columns describe the association results of the human syntenic locus; q-values refer to the marker. All genes that map within 100 kb of the maximum associated marker are listed.

All *suggestive* loci had their syntenic human region tested for genetic association to ethanol-induced anterior-posterior body sway (BSA). Using the comparative genomics functions of Ensembl, all human genes syntenic with the mouse loci were identified. Human subjects had been genotyped previously using the Illumina HumanCNV370-Duo genotyping array. Marker SNPs were chosen for analysis based on location within the genomic limits for the gene’s transcript plus 100 kb flanking the transcription start and stop sites. We thus had markers spanning the transcripts of the syntenic genes plus possible regulatory sequences flanking the transcribed DNA. Each marker SNP was tested for association to BSA using a regression model. False discovery rate q-values were calculated by treating each syntenic locus as a separate hypothesis. The results for each marker are presented in supporting information, Table S1, and the top associated markers are presented in Table 2.

GPC5 (glypican 5) became the gene of interest due to the statistical strength of the linkage data in mice and the association data in humans. The BXD linkage yielded a LOD score of 3.9 (P ≈ 1.25E−05), slightly shy of the genome-wide significance value of 4.3 (P ≈ 5.0E−06). Similarly, the multiple test corrected significance value for association to BSA was q = 0.08 (nominal P = 7.54E−05) very close to the standard q = 0.05 threshold for significance. Taken together, these data implicate GPC5 as a candidate QTL that influences ethanol-induced ataxia.

To further investigate glypican involvement in ethanol response, mutant alleles of the *Drosophila* glypican genes *dally* and *dlp* were investigated to determine whether they alter *Drosophila* ethanol response. The six mammalian glypican genes have two homologs in the *Drosophila* genome. Evolutionarily, *dally* is thought to be orthologous to the human glypican gene paralogs GPC3 and GPC5, whereas *dlp* is orthologous to the other four human glypican paralogs (Filmus *et al.* 2008). We tested both genes in *Drosophila*, reasoning that homologous gene function as measured by something as complex as behavior may not precisely track with the predicted evolutionary lineage of a gene.

**Figure 2:**
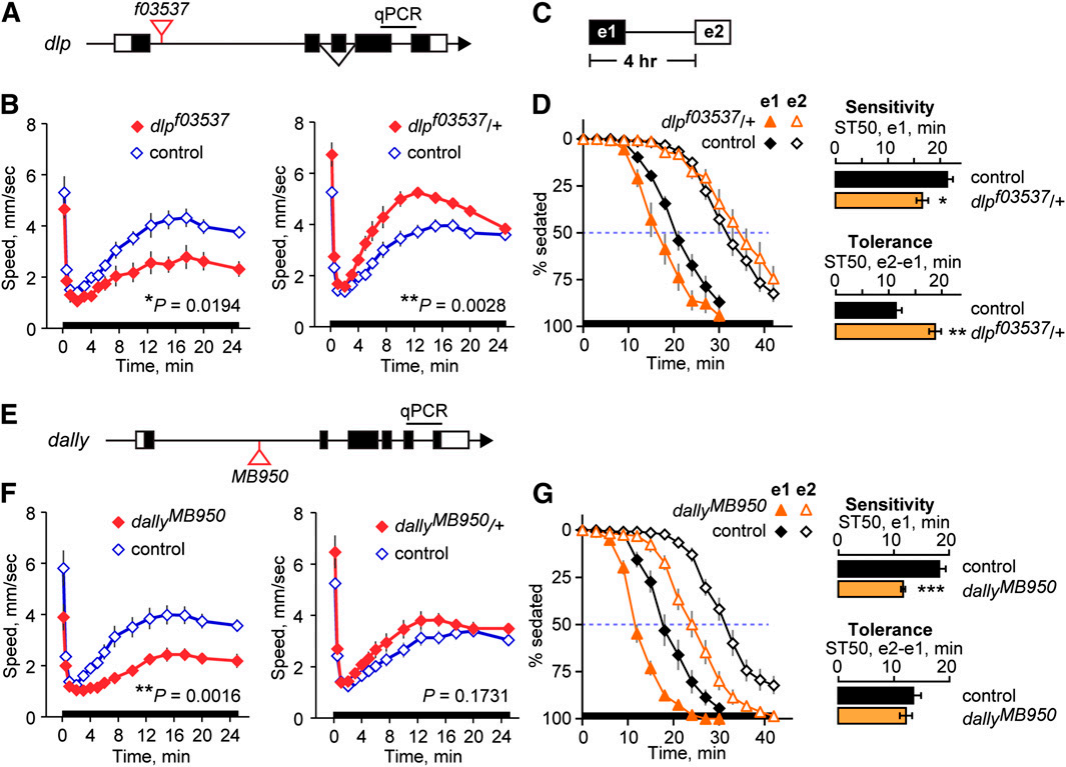
*Drosophila* glypican homologs regulate behavioral responses to acute and repeated ethanol exposure. (A) Simplified diagram of the *dlp* locus. Coding regions are indicated with shaded boxes. The transposon insertion allele *dlp^f03537^* is located in the first intron. Probes for qPCR span the last intron. (B) Ethanol-stimulated locomotion of flies homozygous (left) or heterozygous (right) for *f03537* continuously exposed to 47% ethanol vapor (0–25 min, bar on horizontal axis). Control in all experiments was the genetic background strain. Statistical significance (two-sample *t*-test) was assessed by comparison of the total distance traveled during the hyperactive phase (2–25 min). *f03537*: n = 5, *f03537*/+: n = 8. (C) Exposure scheme for inducing and detecting ethanol rapid tolerance. Flies were exposed twice to 67% ethanol vapor with 4 hr between the start of exposures 1 and 2. (D) Sedation sensitivity and tolerance of flies heterozygous for *f03537*. ST50 is the time to 50% sedation, and sedation tolerance is the difference between the ST50 of exposure 2 (e2) and 1 (e1). Sensitivity and sedation are illustrated by the horizontal bar graphs. ^*^P = 0.0206, ^**^P = 0.0022, two-sample *t*-test. n = 9. (E) Simplified diagram of the *dally* locus. (F) Ethanol-stimulated locomotion of flies homozygous (left) or heterozygous (right) for *MB950* continuously exposed to 47% ethanol vapor (0–25 min). *MB950*: n = 7, *MB950*/+: n = 11. (G) Sedation sensitivity and tolerance of flies homozygous for *MB950*. ^***^P = 0.0002, two-sample *t*-test. n = 7. Ethanol absorption was unaltered in *dlp* (control: 24.6 mM, *f03537*/+: 24.2 mM, *P* = 0.8726, two-sample *t*-test, n = 6) and *dally* mutant flies (control: 29.5 mM, *MB950*: 31.7 mM, *P* = 0.5231, two-sample *t*-test, n = 4).

Partial loss-of-function alleles of *dlp* and *dally* were used in ethanol behavioral assays because they were less afflicted with the developmental defects and lethality caused by strong loss-of-function alleles. Stocks containing partial loss-of-function alleles, either *dlp^f03537^* or *dally^MB950^*, were first outcrossed onto the *Berlin* genetic background to normalize the genetic background between experimental and control flies. Each allele contains a transposon inserted into the first intron (Figure 2, A and E). *dlp^f03537^* was found to be weakly viable and sterile as a homozygote but without overt morphological or behavioral phenotypes. Transcript levels of *dlp* in fly heads were reduced by 40% in *dlp^f03537^* heterozygotes (*P* = 0.0363, n = 3 replicates, two-sample *t*-test); *dlp^f03537^* failed to complement *dlp^1^* lethality, strong evidence that *dlp^f03537^* is a *dlp* allele. *dally^MB950^* was found to be homozygous viable and fertile, and it also had no overt morphological or behavioral phenotypes. Heads of homozygous *dally^MB950^* flies were shown to have a 27% reduction in *dally* transcript (*P* = 0.0218, n = 5 replicates, two-sample *t*-test), but *dally^MB950^* complemented the sterility and wing vein morphology phenotypes of the strong loss-of-function allele *dally^80^*. Given the molecular evidence that *dally^MB950^* is an allele of dally, a transposon inserted into the gene coupled with reduced transcript production, we conclude that intragenic complementation is occurring because the *dally^MB950^* allele provides enough function to complement the phenotype of the strong loss-of-function allele.

Flies carrying the *dally^MB950^* and *dlp^f03537^* alleles were evaluated for alterations in their response to ethanol. Three assays were performed: locomotor stimulation in response to moderate concentrations of ethanol; sedation in response to higher concentrations of ethanol; and the acquisition of rapid tolerance to ethanol sedation. Flies exposed to a continuous stream of moderate concentration ethanol vapor (47%) exhibit a characteristic pattern of locomotor activity. When ethanol is first introduced, flies show a transient burst of locomotor activity—a startle response to the smell of ethanol (Figure 2, B and F). Activity briefly returns to baseline levels, followed by increasing hyperactivity that coincides with the rising internal concentrations of ethanol (approximately 25 mM at 25 min exposure). As internal ethanol concentration increases, flies become progressively more uncoordinated and eventually they become sedated. Sedation sensitivity is measured using a higher ethanol dose, 67% vapor, which results in 50% sedation after ≈20 min exposure in control flies (Figure 2, D and G). If ethanol-exposed flies are allowed to rest for 3.5 hr to metabolize absorbed ethanol, reexposed flies take substantially longer to sedate (≈32 min for 50% sedation). This acquired resistance to ethanol sedation is termed rapid tolerance and is due to neuro-adaptations to the effects of ethanol intoxication (Scholz *et al.* 2000; Wolf and Heberlein 2003).

The ethanol behavioral responses of *dlp^f03537^* and *dally^MB950^* flies differed significantly from controls, and they were qualitatively similar to each other. Ethanol-induced locomotor activity in homozygous mutant flies was the most similar response; both mutant strains, as homozygotes, showed significantly reduced ethanol hyperactivity (Figure 2, B and F, left panels). Interestingly, heterozygous mutants showed the opposite result of increased ethanol hyperactivity, but only *dlp^f03537^* heterozygotes showed a significant increase (Figure 2, B and F, right panels). Given that the expression and complementation experiments indicate that transcript levels decrease with the number of mutant alleles, the simple model that ethanol hyperactivity decreases with decreasing transcript is not supported; a more complex relationship between *dally* and *dlp* gene expression and ethanol hyperactivity must exist. *dally^MB950^* and *dlp^f03537^* mutants were also similar in their ethanol sedation sensitivity. Because of the weak viability of *dlp^f03537^* homozygotes, only heterozygotes were generated in sufficient numbers for the sedation assays. The *dlp^f03537^* heterozygotes displayed increased sedation sensitivity (Figure 2D). *dally^MB950^* heterozygotes and homozygotes were both more sensitive to the sedating effect of ethanol, with the homozygotes exhibiting greater sensitivity (Figure 2G and data not shown). The two mutants differed in their development of rapid tolerance [rapid tolerance was measured as the difference in sedation sensitivity between an initial exposure to ethanol, e1, and a second exposure, e2, delivered four hours later (Figure 2C)]. While *dlp^f03537^* heterozygotes developed greater tolerance, *dally^MB950^* heterozygotes and homozygotes were indistinguishable from control flies (Figure 2, D and G). Ethanol absorption was unaltered in the mutants (Figure 2 legend), so we can conclude that the behavioral alterations were due to different responses to the same ethanol dose.

## Discussion

Our search for genes influencing AUD development first looked for genetic linkage in mice, and we discovered 13 candidate QTL contributing to ethanol-induced ataxia. These hypothetical QTL were examined for genetic associations with alcohol-induced ataxia in humans. With candidate loci as a hypothesis, we reduced the number of statistical tests in comparison with a genome-wide association approach and thus increased the statistical power to detect genetic associations at the human loci. We also obtained greater genetic resolution and human relevance.

Human genetic association identified GPC5, a member of the glypican gene family, to be of statistical interest with a multiple-test–corrected FDR q-value of 0.082. We then moved to a *Drosophila* model system to obtain correlative evidence in an experimental system where mutations can be introduced and tested for their effect on ethanol-induced behaviors. The *Drosophila* results supported the mammalian genetic results by demonstrating that flies carrying mutant alleles of *dally* or *dlp*, the *Drosophila* homologs of mammalian glypicans, exhibit alterations in ethanol-induced locomotor activity, ethanol-induced sedation, and rapid tolerance to ethanol-induced sedation. Together, these results strongly implicate GPC5 as a contributor to ethanol response.

The mammalian glypicans (GPC1 to GPC6) are heparan sulfate proteoglycans (HSPG) linked to the cell surface via a glycosylphosphatidylinositol (GPI) anchor. Much of what we know about glypican function in mammals was founded upon *Drosophila* experiments investigating *dally* and *dlp*, orthologs of the mammalian 3/5 subfamily and the 1/2/4/6 subfamily, respectively [reviews in Filmus *et al.* (2008) and Wu *et al.* (2010)]. Glypicans are involved in modulating cellular signaling, participating in many aspects of normal morphogenesis (including neuronal development and axon guidance) where they regulate the signaling of the Wnt, Hedgehog, fibroblast growth factor (FGF), and bone morphogenetic protein (BMP) ligands. Consistent with this role in developmental signaling, dysregulation of glypicans has been detected in various cancers (Filmus 2001).

GPC5 is developmentally expressed in a pattern consistent with its involvement in central nervous system, limb, and kidney development (Saunders *et al.* 1997). Because acute ethanol response is brain mediated, only brain expression patterns in mice were considered here in detail. Unlike the majority of glypican family members where expression in embryonic brain is much greater than in adult brain, GPC5 brain expression increases with embryonic age and is highest in adult tissue (Luxardi *et al.* 2007). GPC5′s embryonic brain expression is also more spatially restricted than the other glypicans; it is limited to the striatum primordium and ventral diencephalic wall starting about day 10 of embryogenesis (Luxardi *et al.* 2007). In adults, the greatest expression is seen in the caudate nucleus, the putamen, and the hippocampus (Saunders *et al.* 1997), structures thought to play a significant role in drug behavior (Koob and Volkow 2010). In flies, both *dally* and *dlp* are expressed in the nervous system during development and in adulthood (Chintapalli *et al.* 2007).

In addition to being expressed in brain regions thought to play a role in ethanol response, there is evidence that GPC5 transcription can be modulated by ethanol. After discovering that ethanol-induced expression of *Gabra4* in mouse cortical neurons is dependent upon the activation of heat shock factor 1 (HSF1) and its binding to an ethanol response sequence element located between exons 1 and 2, Pignataro *et al.* (2007) performed a microarray screening experiment to identify additional genes whose transcription is activated by ethanol via HSF1. By exposing cortical neurons to ethanol or heat and using microarrays to identify genes whose expression was altered by the treatment, they identified 50 genes that were transcriptionally upregulated in response to ethanol and ∼450 genes upregulated in response to heat. The authors highlighted the 9 genes that responded “dramatically” (upregulated 50% or greater) to both treatments, a list that includes GPC5. Additionally, we previously found that *Drosophila dally* expression was transiently increased following exposure to a sedating dose of ethanol (Kong *et al.* 2010).

Glypicans have been shown to participate in organismal development through their interactions with the Wnt, Hedgehog, FGF, and BMP morphogen signaling pathways (Selleck 2000). These developmental pathways have more recently been implicated not only in neural development but also in postdevelopmental synaptic maintenance and plasticity, making them credible participants in neurological, psychiatric, and behavioral phenotypes (Budnik and Salinas 2011; Farías *et al.* 2010; Ille and Sommer 2005; Marqués 2005; Okerlund and Cheyette 2011; Salinas 2005). We hypothesize that glypicans modulate these pathways in their neurological functions as they do in their developmental functions. Whereas, prior to this report, there was no experimental literature addressing glypicans’ role in behavior, there are reports of glypicans being involved in human psychiatric conditions: GPC6 is associated with a neuroticism using an age-by-SNP interaction model (Calboli *et al.* 2010), and GPC1 is a member of a schizophrenia gene network derived from a gene set enrichment analysis of GWA data (Potkin *et al.* 2010).

In summary, we presented evidence that the glypican GPC5 participates in human ethanol response, an endophenotype related to AUD risk. The strength of the evidence is derived from the coalescence of results across different experimental systems: mouse linkage identified the candidate locus, human genetic association identified a single gene contained within the mouse-defined locus, and *Drosophila* behavioral experiments demonstrated that mutant alleles of the gene identified in mice and humans alter the fly’s ethanol response. In addition to our analyses, the literature on GPC5 reports its expression to be spatially and temporally consistent with a putative involvement in ethanol behaviors, and moreover, its expression can be modulated by ethanol. Finally, the known functions of glypicans as modulators of cell signaling systems that participate in neural development as well as synaptic maintenance and plasticity are also consistent with participation in ethanol-induced behaviors. GPC5 is thus a strong candidate as a gene involved in ethanol response and the development of alcohol use disorders.

## Supplementary Material

Supporting Information
