# Supplementary material for: Glypican Gene GPC5 Participates in the Behavioral Response to Ethanol: Evidence from Humans, Mice, and Fruit Flies
Source: G3 (Bethesda). 2011 Dec 1;1(7):627–35. doi: 10.1534/g3.111.000976 (PMC3276178; doi:10.1534/g3.111.000976)
Supplement: Supporting Information [file supp_1_7_627__index.html]

Supporting Information 

# Glypican Gene GPC5 Participates in the Behavioral Response to Ethanol: Evidence from Humans, Mice, and Fruit Flies

## Supporting Information for Joslyn *et al.*, 2011

**Files in this Data Supplement:**

- Table S1 - Supporting Data (.xls, 1.7 MB)
